# First flush of non-point source pollution and hydrological effects of LID in a Guangzhou community

**DOI:** 10.1038/s41598-019-50467-8

**Published:** 2019-09-25

**Authors:** Jiajun Zeng, Guoru Huang, Haiwan Luo, Yepeng Mai, Haichun Wu

**Affiliations:** 1 South China University of Technology, School of Civil Engineering and Transportation, Guangdong Guangzhou, 510640 China; 2 South China University of Technology, State Key Laboratory of Subtropical Building Science, Guangdong Guangzhou, 510640 China; 3 Guangdong Engineering Technology Research Center of Safety and Greenization for Water Conservancy Project, Guangzhou, 510640 Guangdong China

**Keywords:** Environmental impact, Hydrology

## Abstract

To study the first flush effect of nonpoint source pollution in the Guangzhou community unit, runoff from roads, roofs, and green spaces during three rainfall events was collected and analyzed for pollutants. Nine runoff pollution indices were considered. The dimensionless cumulative curve of pollutant mass vs. volume, the first flush coefficient (*b*) and the mass first flush ratio (MFF_n_) were used to assess the first flush effect of different underlying surfaces. The assessment results pointed out that the roof was most prone to first flush effect. And ammonia nitrogen and phosphorus were the main pollutants in the first flush in the study area. For a quantitative analysis of the first flush, the Storm Water Management Model (SWMM) was used to simulate the hydrological effect of low impact development (LID) implementation in the community. The results showed that the first flush strength was reduced after setting LID. And LID measures, such as green roofs and sunken green spaces, contribute to flood control and rainwater purification. This research can be relevant regarding for constructing sponge cities and reducing the pollution caused by the first flush.

## Introduction

Urban nonpoint source pollution refers to pollutants that flow into rivers and lakes via the runoff formed by rainfall, which then pollute the receiving water in the city. The sponge city, with good resilience in adapting to environmental changes and responding to natural disasters caused by rainwater, is becoming more and more popular in China. The concept of sponge city was proposed in “Sponge City Construction Technology Guide”^1^ to alleviate urban water environmental problems in modern cities such as urban waterlogging, rainwater runoff pollution and water shortage. As sponge city construction in China spreads, investigation of the first flush via rainfall runoff is an important basis for the study of characteristics control of urban nonpoint source pollution. The first flush is the phenomenon that a greater fraction of contaminant is washed out during the initial stage of a rainfall event^2–4^. It is mainly caused by the quick wash off of pollutants accumulated on the watershed surfaces^5^. One of the purposes of sponge city construction is to reduce initial rainwater pollution. The research on first flush effect is an important part of sponge city construction. Bach *et al*.^6^ demonstrated an assessment method to detect the presence and characteristics of the first flush effect. The installation of a device to divert the first flush water away from the collection system may improve the quality of the harvested water^7^. Xiaoping *et al*.^8^ assessed the differences in first flush water quality by designing a rainfall harvesting system to monitor rainwater drained from roofs. Many factors affect the occurrence and intensity of the first flush. Park *et al*.^9^ estimated the probability of the mass first flush ratio (MFF_n_) by using the Storm Water Management Model (SWMM) to analyze the characteristics of the first flush.

In all of these studies, the emphasis is on the analysis of the first flush characteristics without quantitative analysis on the first flush effect. And first flush effect of different underlying surfaces is rarely studied. Then in this research, the first flush of different underlying surfaces is discussed, and quantitative analysis is proposed. Moreover, some measures are proposed to mitigate the degree of contamination. To compare the difference in these methods, three kinds of methods were adopted to evaluate the first flush effect in the present study: The dimensionless cumulative curve called M(V) curve, mass first flush ratio (MFF_n)_ and the first flush coefficient (*b*). The M(V) curve is a qualitative method to evaluate the pollutants load distribution in storm runoff while MFF_n_ is used to quantitatively analyze the amount of pollutant load in runoff. And the value *b* can be used to analyze the first flush strength. Comprehensive analysis of the results using the different methods can be propitious to quantitative analysis of the first flush in the study area and helpful for putting forward a strategy of pollution reduction. Low impact development (LID) system layout may affect the hydrological characteristics significantly, thus further study via SWMM on the efficiency of runoff and pollutant control is still needed^10^. LID system significantly reduced storm-water runoff and runoff quality. In the process of sponge city construction in Guangzhou, the principles and applications of LID have the potential to mitigate urban nonpoint source pollution and the impact of imperviousness in urban areas^11^. Efforts to investigate the effectiveness of LID practices have largely been directed toward microscale evaluation of green roofs, green spaces, permeable pavement, and other LID practices^12^. The reductions in runoff volume and pollutant loads after implementing LID practices have been simulated. The simulation results were compared with the observed impacts and showed the reductions in runoff volume and pollutant loads^13^. Therefore, the simulation results are capable of assisting decision-makers in evaluating the environmental impacts of LID.

In the present study, the authors characterized the first flush effect on roads, roofs, and green spaces in the community of Guangzhou. Different pollutants indices were tested to investigate the influencing factors on the first flush effect. The characteristics of first flush effect in Guangzhou is explored. Pollution caused by first flush is part of urban non-point source pollution. Attention should be paid to the pollution caused by first flush and quantitative analysis of the first flush in Guangzhou according to a comprehensive analysis of different methods. LID design plan is proposed to reduce the first flush pollution. A model of the community unit in Guangzhou was built using the SWMM to analyze the effect of LID. Based on the characteristics of the first flush and the simulation results from the SWMM, suggestions for construction of the sponge city are proposed. The research results provide an important scientific basis for reducing the level of pollutants in the first flush.

**Figure 1:**
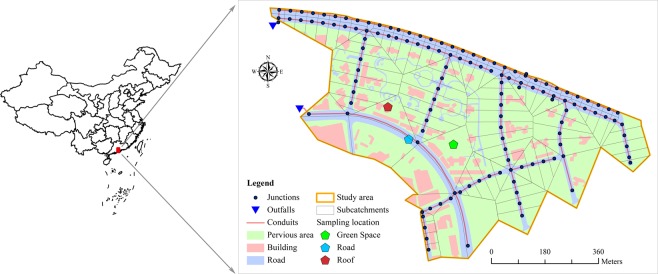
Study area location

The study area is a community located in Guangzhou, China (Fig. 1). It is a housing estate that is an independent closed drainage system, was built in 2013, and covers 432 thousand square meters. The land use of the area is mainly composed of roads, public squares, residential areas, and green spaces.

Three typical underlying surfaces, i.e., roads, roofs, and green spaces, were selected for sampling. When a rainfall event occurred, rainfall runoff samples were collected manually every 10 minutes at different sampling sites and recording each sampling time. Three different bottles were filled at each sampling site. The samples were used to analyze different types of underlying stormwater runoff pollutant types five-day biochemical oxygen demand (BOD_5_), chemical oxygen demand (COD_Cr_), total suspended solids (TSS), ammonia nitrogen (NH_3_-N), total nitrogen (TN), and total phosphorus (TP). As the properties of COD_Cr_, NH_3_-N, TN, and TP will change over time and the sampling sites are far from the laboratory, concentrated sulfuric acid was added to the samples immediately after collection to avoid changes in these indicators. The A and B bottles were used for the determination of BOD_5_ and TSS, respectively, and the C bottles were used for determining the COD_Cr_, NH_3_-N, TN, and TP, thus concentrated sulfuric acid was added into the C bottles.

In this study, the authors collected samples during three rainfall events on September 2, 7, and 10 in 2016. The rainfall characteristics of rainfall duration (*T*_*r*_), rainfall (*W*), average rainfall intensity (*I*_*a*_), and maximum rainfall intensity (*I*_*max*_) for the three events are shown in Table 1. There’s no rain a week before September 2th and no other rain between the three rainfall events.

**Table 1:** Rainfall characteristics of the three rainfall events.

Date	T_r_/[min]	W/[mm]	I_a_/[mm·h^−1^]	I_max_/[mm·h^−1^]
Sep 2^nd^	37	6.3	8.1	24
Sep 7^th^	90	10.1	3.4	18
Sep 10^th^	66	8.1	1.0	13

Samples(N = 52) were tested according to the methods listed in Table 2. The maximum concentration *C*_*max*_, the median concentration *C*_*med*_, the minimum concentration *C*_*min*_, the average concentration $\overline{C}$, the standard deviation *σ*, and the coefficient of variation *C*_*v*_ of each pollution indicator in the samples are shown in Table 3. Statistics from the test results indicate that the greatest variation occurred for BOD_5_, COD, and TSS, and the least variation occurred for TN.

**Table 2:** Test methods of each index.

Test items	Test methods
BOD_5_	Dilution and inoculation HJ 505-2009
COD_cr_	Dichromate method GB/T 11914-1989
TSS	Gravimetric method GB/T 11901-1989
NH_3_-N	Sodium Reagent Spectrophotometry HJ 535-2009
TN	Alkaline potassium persulfate digestion UV Spectrophotometry HJ 636-2012
TP	Ammonium molybdate spectrophotometric method GB/T 11893-1989

**Table 3:** Summary statistics (N = 52) for the water quality indicators.

Water quality index	BOD_5_	COD_cr_	TSS	NH_3_-N	TN	TP
*C*_max_ (mg/L)	84.20	393.00	132.00	2.27	3.96	0.26
*C*_med_ (mg/L)	3.75	16.50	8.50	0.62	2.46	0.05
*C*_min_ (mg/L)	1.40	5.00	1.00	0.11	1.35	0.02
$\overline{C}$(mg/L)	7.80	33.80	19.80	0.75	2.43	0.08
*σ*	13.40	63.20	30.10	0.56	0.68	0.06
*C*_*v*_	1.72	1.87	1.52	0.74	0.28	0.73

## Results

### First flush analysis

**Figure 2:**
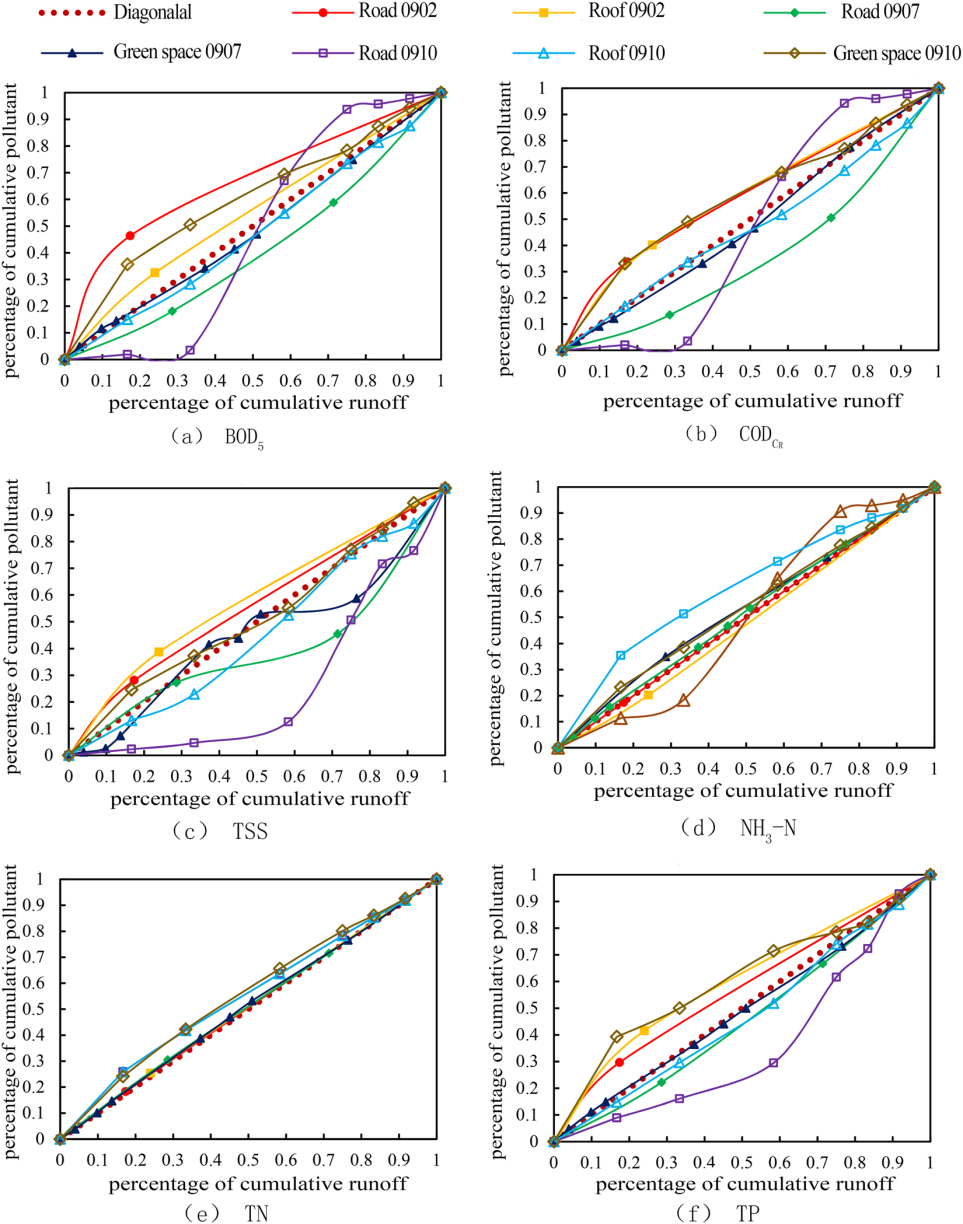
M (V) curves of different pollutions

The M(V) curves for pollutant indices were drawn based on the measured rainfall data and pollutant concentration data, as shown in Fig. 2. The M(V) curves were plotted to represent the normalized cumulated runoff volume for each storm event and each water quality index^14^. If the curve is close to the diagonal line, it indicates that the pollutant concentration changed little during the early stage of runoff or that the load distribution is comparatively uniform^15^.

As illustrated in Fig. 2, the M(V) curves of the pollutant indices all fluctuated in different degrees. Among them, the upward convex part of the curve of BOD_5_, NH_3_-N and TP is more obvious and that indicated that the first flush effect of these pollutant indices is more significant. The curves of TN are the closest to the diagonal line and the curves of TSS are concave, so the first flush effect of TN and TSS is weak. On the other hand, during the first rainfall (Sep/02/2016), the curves of pollutants indices such as BOD_5_, COD_Cr_, TSS, TN, and TP showed that the first flush occurred on the roads. Whereas during the third rainfall (Sep/10/2016), the first flush effect was not obvious for the roads and roofs. The curves for the pollution on the road demonstrate large variability in the strength of the first flush effect. In addition, the M(V) curves of the multiple pollution indices showed the first flush effect of the roof occurred and the strength of first flush for roof is stronger than road and green space. From the above, the first flush effect is most pronounced on the roof and the strength of first flush is most uncertain on road. The main pollutions in first flush are BOD_5_, NH_3_-N and TP.

**Figure 3:**
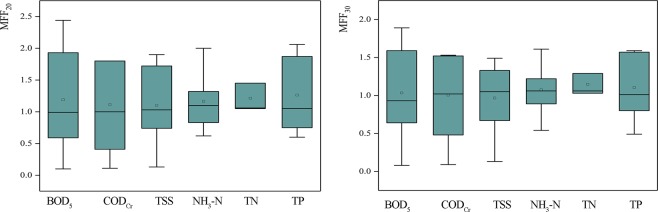
MFF_20_ and MFF_30_ of the different pollutants indices (The base of box denotes the first quartile (25%), the line in the central part of the box indicates the median (50%), and the roof marks the third quartile (75%). The mark in the middle of the box indicates the average. The upper and lower ends of the whiskers denote the Maximum and minimum values.)

Using Eq. (3), the MFF_20_ and MFF_30_ of each pollutant index were calculated and the results are summarized as boxplots in Fig. 3. Figure 3 indicates that for all the contaminants the mean values were near 1.0. Variances for BOD_5_ and COD were about three times the variances for NH_3_-N and TN. The average of the MFF_20_ values are all greater than 1.0, whereas the average of the MFF_30_ values of TSS and COD_Cr_ are less than 1.0. From the general trend, the MFF_20_ values of different pollutants indices are greater than the MFF_30_ values. The greater the value of MFF_n_ the more obvious the first flush effect is. And the MFF_20_ values is average of fourteen points higher than MFF_30_. According to the definition of MFF_n_, the first flush effect is more likely to occur during the first 20% runoff than the first 30% runoff for the community unit in Guangzhou. According to the MFF_20_ calculation results, 100% of the TN indices; 42.9% of the BOD_5_, COD_Cr_, and TSS indices; and 57.1% of the NH_3_-N and TP indices occurred first flush effect.

**Figure 4:**
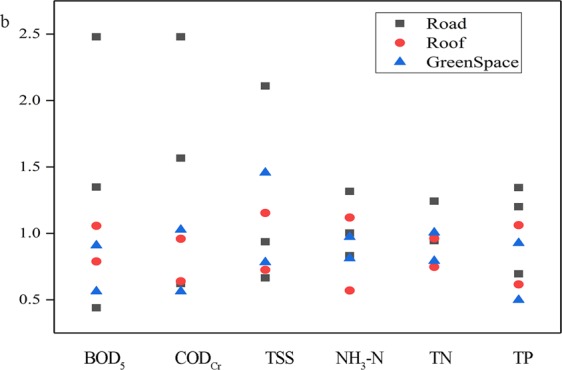
The calculation results of *b* values

Based on Eq. (4), the water quality data were fitted using power function regression and the first flush coefficient (*b*) was calculated. The fitting correlation coefficients were all greater than 0.93, which indicated that the fitting effect was good and the results were suitable for first flush analysis. Figure 4 shows a comparison of pollutant indices on different underlying surfaces and a comparison of first flush effect for different pollutant indices. According to the definition of the value *b*, when *b* < 1.0, the first flush occurred, and the smaller the *b* value, the greater the first flush intensity. Figure 4 shows that 59.5% of the *b* values are less than 1.0. The change ranges of *b* values for NH_3_-N, TN and TP are smaller than the other indices. The intensity of first flush for NH_3_-N and TP are greater because the *b* values are smaller. It indicates that ammonia nitrogen and phosphorus are the main pollutants in the first flush effect of the community unit in Guangzhou. In addition, *b* values for the roof indicate the first flush effect is the most pronounced and *b* values for the road indicate the first flush strength is uncertain. The first flush strength of the green space is weakest. There consist with the result of M(V) curve.

Different methods were used to determine whether the first flush occurred. The results obtained from different methods were generally the same, but not completely consistent. The difference in results illustrates the complexity and uncertainty of the influence factors of the first flush effect.

**Figure 5:**
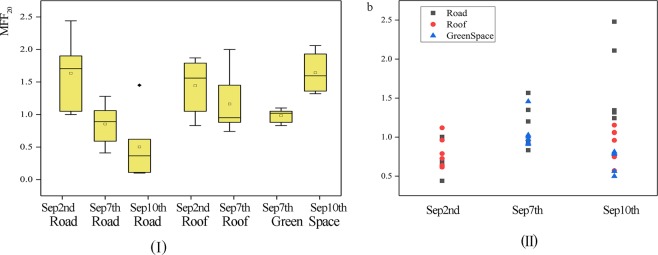
(I) MFF_20_ for the different underlying surfaces. (II) The *b* values for the different rainfall events.

In order to analyze the influence factors of the first flush effect, pollutants are analyzed in different rainfall events. MFF_20_ and first flush coefficient (*b*) were calculated to compare the first flush effect during each rainfall event and some similar results can be found in Fig. 5 (I) and (II). Figure 5 (I) shows that the first flush effect occurred on a majority of the roads and roofs during the first rainfall (Sep 2nd) of which the intensity is the maximum of the three rainfall events. The number of first flushes occurring is less for the second rainfall (Sep 7th) whereas the intensity is weaker than the first rainfall event. Figure 5 (II) shows that the intensity of the first flush effect is the greatest during the first rainfall which indicated that the intensity of rainfall is an influence factor of the first flush. Besides, the first flush effects for roofs are more intensity than that for roads. In addition, the intensity and the occurrence frequency of the first flush of the green spaces during the second rainfall (Sep 7th) are more than during the third rainfall (Sep 10th). This supported the previous conclusion that green spaces function as pollutant interceptors.

### Hydrological effect analysis

There is great uncertainty about the first flush of roads according to the above analysis of the first flush. This may be because the sanitation department is washing the streets daily. Thus, the measures to cut pollution should be concentrated on the roofs and green spaces. The functions of the LID mainly are infiltration, stagnation, storage, purification, usage, and drainage, which can reduce the total amount of runoff discharge and treat the rainwater^16^. Referring to the Sponge City Development Technical Guide compiled by the Ministry of House and Urban-Rural Development of the People’s Republic of China^1^, 50% of the roofs and green spaces were selected for transformation based on the corresponding LID measures. In other words, 50% of the roofs were transformed into green roofs and 50% of the green spaces were transformed into sunken green spaces. Rainwater on the roads and roofs will flow into the sunken green spaces and then flow to the water outlets.

**Figure 6:**
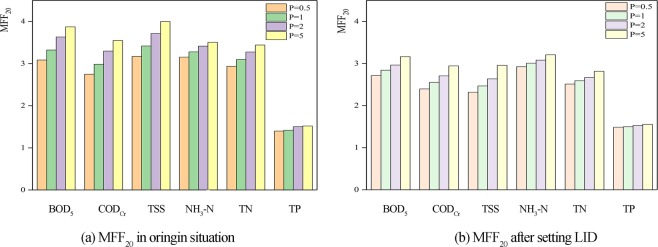
MFF_20_ during different rainfall recurrence period.

According to the flow process of drainage outlets in the simulation results, the MFF_n_ of different pollutants can be calculated. As the first flush effect is more prone to occur during the first 20% of runoff in the above research, the MFF_20_ values were calculated. The situation after the implementation of the LID measures is compared with the original situation in Fig. 6.

The results of the comparison show that the MFF_20_ values of the pollutant indices, decreased after the implementation of the LID measures. In other words, pollutants were reduced in the first 20% of the rainwater runoff. The reduction effect in different rainfall return periods is similar. LID measures such as green roofs and sunken green spaces have an interception effect on pollutants. The decline in MFF_20_ of TP is not obvious after the implementation of the LID measures, whereas, for the whole runoff process, the reduction in TP reached 48%. In general, green roofs and sunken green spaces can lower the degree of pollution caused by the first flush.

## Discussion

The phenomenon of first flush is influenced by many factors. The first flush effects on different underlying surfaces are varied. The first flush strength of the roof and road is stronger than that of the green space. This conclusion is similar previous studies which concluded that the strength of first flush is stronger in the impervious areas^17^. The first flush occurs on the road showing uncertainty, probably because of the reduction of pollutants accumulation after street cleaning. Nitrogen and phosphorus are mainly pollutants in the first flush. The results of nonpoint source pollution studies in adjacent areas^18^ show that nitrogen and phosphorus pollutant load in first flush accounted for more than 50%. However, the factors affecting the first flush effect are various and the first flush strength varies greatly in different regions.

Table 4. shows the analysis results of water quantity and pollutant reduction. The reduction effect on water and pollutants decreases with the increase in rainfall recurrence period. The effect of LID measures is more significant for the low recurrence period rainfall. The reduction effect is positively related to the water storage capacity of LID measures. Because the water storage capacity of LID measures is limited, reduction effect decreased with greater rainfall. The results correctly reflected the objective law that the larger the rainfall, the smaller the reduction rate. According to the results, the reductions in water quantity are more than 43%, which indicated the LID measures had good water storage capacity and a positive effect on flood control. In addition, the LID measures can reduce pollutants in runoff. The reductions of TP are more than 48%, which is the most significant reduction effect among the pollutants. The results from the SWMM simulation show that LID measures contribute to flood control, treat rainwater, and reduce the level of pollutants in the first flush.

**Table 4:** Water quantity and pollutants reduction (%) effect of LID measure.

Recurrence period (a)	Water quantity	BOD_5_	COD_Cr_	TSS	NH_3_-N	TN	TP
P = 0.5	46	38	33	33	37	40	50
P = 1	45	35	32	29	35	37	50
P = 2	44	30	29	23	32	34	49
P = 5	43	23	24	15	29	28	48

Some suggestions can be made for sponge city construction according to the study results. Firstly, rainwater from roofs and roads can be drawn into the green spaces and purification measures can be installed in the green spaces. Green spaces can reduce the direct discharge of polluted water into receiving waters. Secondly, treatment measures for nitrogen and phosphorus pollutants should be considered during sponge city construction. In future studies, the reduction of pollutants will be the focus and more collection of samples during more rainfall events will be the next step.

## Methods

### First flush effect

Early understanding of the first flush effect regarded as the concentration of pollution during initial rainfall. The view was that the concentration of pollutants in early rainfall runoff is higher than that in later stages of runoff. However, the influence of rainfall intensity and runoff cannot be neglected. On one hand, the greater the rainfall intensity, the greater the impact intensity on the surface and the more likely that pollutants are transferred from the underlying surface to the surface runoff^19^. On the other hand, on the premise of the same rainfall intensity and total amount of pollutants, when the runoff is large, the pollution concentration decreases. Because the definition of the first flush is controversial, the M(V) curve, MFF_n_, and first flush coefficient were used to analyze the first flush effect of the study area. The purpose is to conduct a comprehensive quantitative analysis of the first flush effect.

The M(V) curve is commonly used for studying the first flush. The view is that when the accumulative transport rate of pollutants is larger than that of runoff accumulation in the early stage of rainfall, the first flush exists^20^. In other words, a small proportion of total runoff transfers a large proportion of the pollution during a rainfall event. To compare the variation in pollutant loads during rainfall events, M(V) curves can be obtained using Eq. (1) and Eq. (2):

1 $$M(t)={\int }_{0}^{t}Q(t)C(t){\rm{d}}t/{\int }_{0}^{T}Q(t)C(t){\rm{d}}t\approx \mathop{\sum }\limits_{i=0}^{k}\overline{Q}({t}_{i})\overline{C}({t}_{i})\Delta t/\overline{Q}({t}_{i})\overline{C}({t}_{i})\Delta t$$

2 $$V(t)={\int }_{0}^{t}Q(t){\rm{d}}t/{\int }_{0}^{T}Q(t){\rm{d}}t{\int }_{0}^{T}Q(t){\rm{d}}t\approx \mathop{\sum }\limits_{i=0}^{k}\overline{Q}({t}_{i})\Delta t/\mathop{\sum }\limits_{i=0}^{n}\overline{Q}({t}_{i})\Delta t$$

where *M*(*t*) is the ratio of cumulative runoff to total runoff during the rainfall at time *t*; *V*(*t*) is the ratio of cumulative pollutant load to total pollutant load during the rainfall at time *t*; *Q*(*t*) is the instantaneous flux at time *t*, [L/min]; *C*(*t*) is the instantaneous pollutant concentration at time *t*, [mg/L]; *T* indicates the time from which rainfall runoff begins and ends at the end of the runoff, [min]; Δ*t* is the time increment of the calculation, [min]; $\overline{Q}({t}_{i})$ is the average runoff during Δ*t* at time *t*_*i*_, [L/min]; and $\overline{C}({t}_{i})$ is the average value of the pollutant concentration during Δ*t* at time *t*_*i*_, [mg/(L·min)].

According to Code for Design of Outdoor Wastewater Engineering^21^, the fixed runoff coefficient method was used to calculate the runoff via the rainfall data. According to the code^21^, when the catchment areas are not exceeded 2 square kilometers, the runoff can be calculated by inference formula. Then, the average runoff can be calculated using Eq. (3) that the Code recommends:

3 $$\overline{Q}({t}_{i})=\overline{i}({t}_{i})\psi F$$

where $\overline{i}({t}_{i})$ is the average rainfall intensity during Δ*t* at time *t*_*i*_, [mm/min]; *ψ* is the runoff coefficient, and *F* is the catchment area, [hm^2^].

Taking the ratio of cumulative runoff to total runoff as the abscissa and the ratio of pollutant cumulative load to total pollutant load as the ordinate, the dimensionless M(V) curve was plotted. The 45° angle bisector of the coordinate system was used as a standard indicator of the occurrence of the first flush effect. When the M(V) curve lies above the 45° angle bisector, the first flush occurs. On the contrary, if the M(V) curve lies below the 45° angle bisector, the first flush does not occur.

On the basis of M(V) curve, for qualitatively describing the first flush effect, Saget *et al*.^22^ defined that when at least 80% of the pollutant mass is transported in the first 30% of the runoff volume, the first flush exists. Based on the dimensionless cumulative M(V) curve, Ma *et al*.^23^ suggested the MFF ratio, which can be used for quantitative analysis of the first flush effect. The MFF_n_ is the ratio of the percentage of cumulative pollution load to percentage of cumulative runoff when the cumulative transport runoff accounts for *n*% of the total runoff. The calculation formula is shown as Eq. (4):

4 $${{\rm{MFF}}}_{n}=\frac{{\int }_{0}^{t}Q(t)C(t){\rm{d}}t/M}{{\int }_{0}^{t}Q(t){\rm{d}}t/V}\approx \frac{\mathop{\sum }\limits_{i=0}^{k}\overline{Q}({t}_{i})\overline{C}({t}_{i})\Delta t/M}{\mathop{\sum }\limits_{i=0}^{k}\overline{Q}({t}_{i})\Delta t/V}$$

where *n* is the percentage of cumulative runoff to total runoff from the time of runoff generation to time *t*, ranging from 0 to 100%. *M* is the total amount of pollution load during the whole rainfall process, [mg]; and *V* is the total amount of runoff discharged during the whole rainfall process, [L]. The MFF ratio is a method derived from the M(V) curve. According to the previously mentioned about the definition of MFF, 80% of the pollutant mass is transported in the first 30% of the runoff, which means MFF_30_ = 2.67. When the M(V) curve lies above the 45° angle bisector, MFF_n_ >1.0.

This method describes the occurrence conditions of the first flush effect. To analyze the first flush intensity quantitatively, Bertrand-Krajewski *et al*.^24^ demonstrated that there is a power function relationship between the cumulative pollution load percentage and the cumulative runoff percentage, as follows:

5 $$G={L}^{b}$$

where *G* is the proportion of cumulative pollution load and values range from 0 to 1, and *L* is the proportion of cumulative runoff. When *L* = 0, *G* = 0 and when *L* = 1, *G* = 1. The value *b* is the first flush coefficient, which reflects the intensity of the first flush according to the deviation between curve and diagonal. When the M(V) curve lies above the 45° angle bisector, the value of *b* is less than 1.0; the smaller the *b* value, the greater the first flush intensity. A dilution effect was defined for when *b* is greater than 1.0^25^.

### Storm water management model

The SWMM is a widely used stormwater simulation model that was developed for the Environmental Protection Agency^26^. The model can be used for a single event or long-term simulation of runoff quantity and quality from urban areas. LID measures have been implemented in SWMM to simulate the hydrologic performance. And sunken green spaces, green roofs, and permeable pavement have been chosen in this research. LID measures can be assigned within the selected subcatchments and defined the corresponding areal coverage. Basing on permeation theory, Horton infiltration model has been used, and different parameters of structural layers were set correspond to different measures^27^.

In the current research, the drainage system subcatchments of the study area were divided using the equal angle line method. Then, the Tyson polygon rule was used to subdivide the subcatchments based on the locations of manholes. Finally, the study area was divided into 132 subcatchment areas. The water quantity parameters of the model (Table 5) were determined by referring to the model’s manual^26^ and the relevant research on the same study regions^28^. The runoff from 20 rainfall events were monitored and Zhu *et al*.^28^ used the Nash–Sutcliffe efficiency (NSE) index and Kling–Gupta efficiency (KGE) to evaluated the model and suggested that the parameter values were reasonable because the KGE and NSE between the observed runoff timeseries and the simulated data were up to 0.7. Simulated values can be regarded as satisfactory when NSE > 0.5. And simulated values could be regarded as satisfactory with a KGE value as low as 0.6.

**Table 5:** Water quantity parameters of the model.

Parameter	Physical meaning	The recommended parameter range^26^	The parameter of this model
N-Imperv	Manning coefficient of impervious area	0.010–0.015	0.015
N-Perv	Manning coefficient of permeable area	0.012–0.8	0.032
Destore-Imperv	Depth of water storage in impervious area (mm)	0.1–2	1
Destore-Perv	Depth of water storage in permeable area (mm)	2–15	10
% Zero-Imperv	Proportion of no depressions and impervious zones in impervious area	25–100	50
MaxRate	Maximum infiltration rate (mm·h^−1^)	45–120	103.81
MinRate	Minimum infiltration rate (mm·h^−1^)	0–25	11.44
Decay	Osmotic attenuation coefficient	2–7	2.75

The water quality parameters were calibrated using the rainfall event of September 10. Then, the observed data of the September 2 and 7 rainfall events were used to assess the model. Method of trial and error was used and the parameters were adjusted by one-at-a-time method. The correlation coefficient between the simulation results and the observed data was used to characterize the trends of concentration change for pollutants. The water quality model parameters were adjusted until the correlation coefficient is greater than 0.5 and simulated values could be regarded as satisfactory. The final determined water quality parameters are shown in Table 6.

**Table 6:** Water quality parameters of the SWMM model.

Underlying surface types	Parameter	BOD_5_	COD_Cr_	TSS	NH_3_-N	TN	TP
Road	Maximum accumulation /(kg.hm^−2^)	80	300	100	2	5	1
	Accumulation time of semi saturation /d	3~5	3~5	3~5	3~5	3~5	3~5
	Flushing coefficient	0.005	0.007	0.03	0.015	0.02	0.0045
	Flush index	1.8	1.5	1.2	1.8	1.6	1.1
Roof	Maximum accumulation /(kg.hm^−2^)	10	8	10	2	7.5	0.3
	Accumulation time of semi saturation /d	3~7	3~7	3~7	3~7	3~7	3~7
	Flushing coefficient	0.0032	0.03	0.014	0.014	0.006	0.0022
	Flush index	1.4	1.2	1.2	1.8	1.2	1.05
Green space	Maximum accumulation /(kg.hm^−2^)	20	40	80	4	8	2
	Accumulation time of semi saturation /d	3~20	3~20	3~20	3~20	3~20	3~20
	Flushing coefficient	0.006	0.03	0.024	0.008	0.011	0.0009
	Flush index	1.8	1.5	1.6	1.25	1.42	1.6

The simulation of the transformation plan was completed in the SWMM, and the effect of LID measures under various rainfall recurrence periods was analyzed. The rainstorm intensity in each recurrence period was calculated by using the rainstorm intensity formula of Guangzhou and the design rainfall hydrograph using the Chicago rainfall pattern which is determined according to a rainfall pattern using the Chicago Hydrograph Model^29^. The water quality parameters of the model were calibrated and assessed using the sample data of the three rainfall events.
